# Lack of Association between Genetic Polymorphisms of JAK-STAT Signaling Pathway Genes and Acute Anterior Uveitis in Han Chinese

**DOI:** 10.1155/2016/5896906

**Published:** 2016-11-14

**Authors:** Ling Cheng, Hongsong Yu, Yan Jiang, Juan He, Sisi Pu, Xin Li, Li Zhang

**Affiliations:** The First Affiliated Hospital of Chongqing Medical University, Chongqing Key Laboratory of Ophthalmology and Chongqing Eye Institute, Chongqing, China

## Abstract

*Purpose.* This study aimed to investigate the association between single nucleotide polymorphisms (SNPs) of JAK-STAT signaling pathway genes and acute anterior uveitis (AAU) with or without ankylosing spondylitis (AS) in the Han Chinese population.* Methods*. Eleven SNPs of the* JAK1*,* JAK2*,* STAT1*,* IRF1*, and* NOS2* genes were analyzed in 443 AAU patients with AS, 486 AAU patients without AS, and 714 healthy controls. Genotyping was performed by PCR-RFLP assay or TaqMan® probe assay. The Chi-squared (*χ* ^2^) test and multivariate logistic regression analysis were used to compare the distributions of alleles and genotypes between patients and controls. *P* values were adjusted using Bonferroni correction.* Results*. We did not observe significant differences in the genotype and allele frequencies of any SNP between AAU patients with or without AS and healthy controls. Stratification analyses by gender and HLA-B27 status showed a boundary significant association between two SNPs (rs10975003 and rs10758669) in* JAK2* and AAU (*P* = 0.052 and *P* = 0.053, resp.).* Conclusions*. Our results indicated that genetic polymorphisms of the JAK-STAT signaling pathway genes may not be associated with AAU in the Han Chinese population.

## 1. Introduction

Uveitis is one of the major ocular diseases leading to blindness and visual impairment. The prevalence of uveitis is 111.3 per 100,000 persons in Taiwan [1] compared with 40.4 per 100,000 persons in Japan [2] and 115.3 per 100,000 persons in United States [3]. In the clinic, acute anterior uveitis (AAU), which may be accompanied by complicated phenotypes including cataract and glaucoma [4], is the most common type of uveitis [5]. Evidence suggests that the occurrence of AAU is associated with the prognosis of ankylosing spondylitis (AS) [6, 7]. The frequency of AAU, which is characterized by positive human leukocyte antigen- (HLA-) B27, varies across different ethnic populations [8–10]. In the United States and Western Europe, the prevalence of HLA-B27 with AAU is up to 50% [5, 8, 11]. Previous studies have reported that there is a strong association between AS and HLA-B27 in various ethnic groups [12–14]. Further study showed that the percentage of AAU accompanied by AS is 30–40%, suggesting that there may be linked pathogenesis between AAU and AS [15]. AAU and AS may share certain genetic associations, but several susceptibility genes seem to be unique for each disease [16]. Genes including* TNFSF15*,* TRAF5*, and* FoxO1* have been reported to be associated with AAU [17–19]. However, a lack of association with AAU has been demonstrated for other genes, including* CTLA4* and* PTPN22* [20, 21]. A recent study revealed that T lymphocyte subsets (Th1 and Th17) and CD4^+^ CD25^+^ Treg cells were involved in the development of HLA-B27 positive AAU [22, 23]. Furthermore, a higher level of Th17 cells has been observed in the peripheral blood of patients with AS [24].

The Janus kinase-signal transducer and activator of transcription (JAK-STAT) signaling pathway plays a major role in T lymphocyte differentiation and function [25, 26].* JAK1* and* JAK2* have been reported to play an important role in Th1 and Th17 cell differentiation [27].* STAT1* is critical to T lymphocyte differentiation and function [25, 26, 28].* STAT1* is activated by type I interferons (IFNs) and IFN-*γ* and plays an important role in immune responses [29].* IRF1 *is the first member identified in the IRF family and is involved in many innate and adaptive immune responses [30]. Impaired or absent Th1-type immune responses favor Th2 differentiation in* IRF1*-deficient mice [31, 32].* NOS2*-derived NO, a key factor in immunoregulation [33], can inhibit Th1 as well as Th2 cytokine production and regulate the development of FoxP3^+^ Treg cells [34, 35]. In summary, JAK-STAT signaling pathway genes, including* JAK1, JAK2, STAT1, IRF1*, and* NOS2*, have been suggested to be strongly linked with T cells and may be involved in the pathophysiology of AAU with or without AS.

Thus, we conducted the present case-control study to investigate whether JAK-STAT signaling pathway genes confer susceptibility to AAU risk in a Chinese Han population.

## 2. Materials and Methods

### 2.1. Subjects

A total of 929 AAU patients were enrolled in this study, including 443 patients with AS (AAU^+^AS^+^) and 486 patients without AS (AAU^+^AS^−^), as well as 714 gender- and race-matched healthy controls. All subjects were Han Chinese recruited from the Department of Ophthalmology in the First Affiliated Hospital of Chongqing Medical University (Chongqing, China) between June 2008 and May 2015. All AAU patients were diagnosed based on medical records, physical examinations, and the anatomic location of inflammation as previously described by Jabs et al. [36]. The diagnosis of AS followed the modified New York Criteria [37]. All subjects gave written informed consent before blood collection. This study was approved by the Human Ethics Committee of the First Affiliated Hospital of Chongqing Medical University (Approval number: 2009-201008) and followed the tenets of the Declaration of Helsinki.

### 2.2. SNP Selection

**Figure 1:**
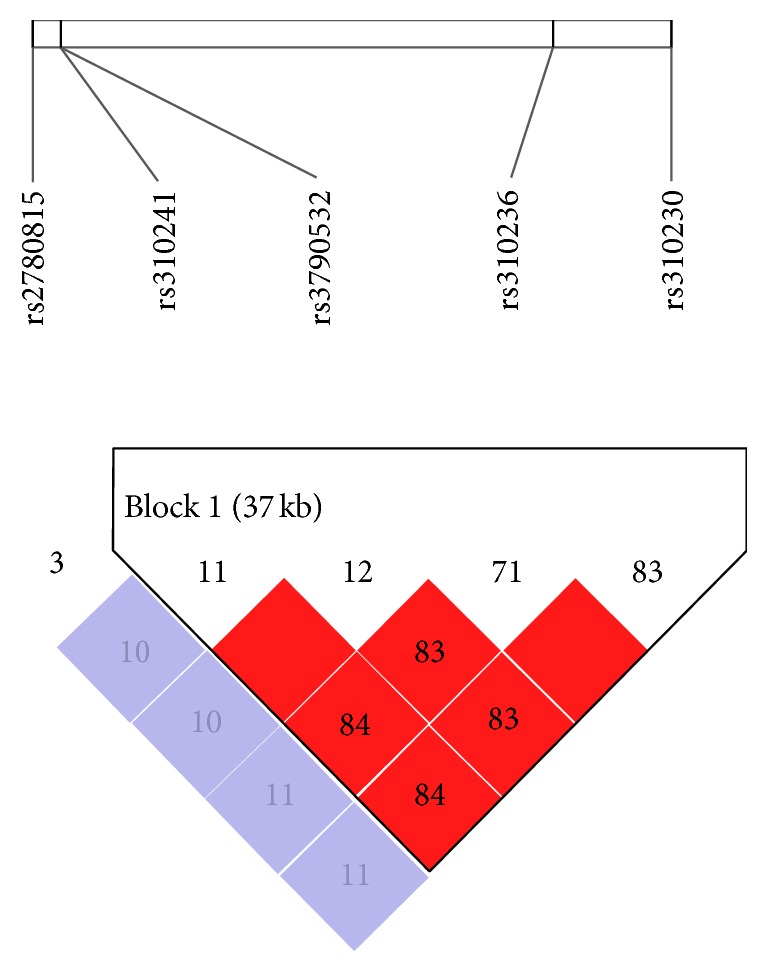
Linkage disequilibrium (LD) analysis of rs2780815, rs310241, rs3790532, rs310236, and rs310230 in the* JAK1 *gene. The LD block was estimated by HaploView software version 4.2 using the Chinese Han HapMap data. The number in the square indicates the *r* ^2^ value.

We selected candidate single nucleotide polymorphisms (SNPs) based on previously published studies and included only those SNPs significantly associated with autoimmune diseases [38–45]. We used HaploView 4.2 software to evaluate the linkage disequilibrium (LD) and minor allele frequency (MAF) of the SNPs. Five SNPs of* JAK1*, rs2780815, rs3790532, rs310230, rs310236, and rs310241 [41, 42], were selected. Since the SNPs rs3790532, rs310230, rs310236, and rs310241 are in strong LD with each other (*r* ^2^ > 0.8, Figure 1), we only used rs310241 in our study. Furthermore, we also eliminated SNPs that were not polymorphic in the Chinese population. Finally, eleven SNPs in five JAK-STAT signaling pathway genes were tested in our study, including two SNPs in the intron region of the* JAK1* gene (rs310241, rs2780815) [41], two SNPs in the exon region and 3′UTR of the* JAK2* gene (rs10758669, rs10975003) [38, 45], one SNP in the intron region of the* IRF1 *gene (rs2070721), four SNPs in the exon region and intron region of the* STAT1* gene (rs2066802, rs1547550, rs6718902, and rs10199181) [39, 40], and two SNPs in the exon region and intron region of the* NOS2* gene (rs2297518, rs4795067) [43, 44].

### 2.3. DNA Extraction and Genotyping

Peripheral blood samples were collected from subjects, and genomic DNA extraction was performed using the QIAamp DNA Blood Mini Kit (Qiagen, Valencia, CA, USA). The genomic DNA was quantified with NanoDrop 2000 (Thermal Fisher Scientific, Delaware, USA) and stored at −20°C until use. Three SNPs (rs2780815, rs2070721, and rs10199181) were genotyped by TaqMan probe (Applied Biosystems, Foster City, CA), and the others were genotyped by polymerase chain reaction restriction fragment length polymorphism (PCR-RFLP) assay. The specific primers for PCR and restriction enzymes are described in Table 1. The PCR reactions were performed under the following conditions: denaturation at 95°C for 5 minutes, 33 to 36 cycles of denaturation at 95°C for 30 seconds, annealing at 56–64°C for 30 s, and extension at 72°C for 30 seconds and a final extension at 72°C for 5 minutes. The enzyme-digested products were visualized on 3% or 4% agarose gels and stained with GoldView (SBS Genetech, Beijing, China). Direct sequencing was carried out randomly on 10% of the study samples to assure the validity of the SNP genotyping method used. The success rate of all SNP genotyping ranged from 97.3% to 100%.

### 2.4. Statistical Analysis

Hardy-Weinberg equilibrium (HWE) was analyzed by the *χ* ^2^ test. The distributions of the allele and genotype frequencies between the patients and controls were compared by the *χ* ^2^ test. Multivariate logistic regression model adjusted for age and gender was further adopted to test the associations between the SNPs and AAU. The risk effect of each SNP was measured by odds ratios (OR) and 95% confidence intervals (CI). There were four different models of inheritance in our study, including additive models, codominant models, dominant models, and recessive models. *P* values were corrected using the Bonferroni correction method considering multiple tests. Statistical significance level was defined as a corrected *P* value < 0.05. Statistical analyses were performed using SPSS version 17.0 (SPSS, Inc., Chicago, IL, USA).

## 3. Results

### 3.1. Clinical Features of AAU Patients

The detailed clinical features and demographic characteristics of the AAU patients are presented in Table 2. All 929 AAU patients include 569 (61.2%) males and 360 (38.8%) females. The 714 control subjects consisted of 428 (59.9%) males and 286 (40.1%) females. The average age was 39.8 ± 12.3 in AAU patients and 39.5 ± 10.8 in the controls, respectively. There were no significant differences in age and gender between the cases and controls. In addition, 546 AAU patients (68.9%) were HLA-B27-positive, whereas 246 AAU patients (31.1%) were HLA-B27-negative.

### 3.2. The Genotype and Allele Frequency Distribution of the Tested SNPs in AAU

Eleven SNPs of the JAK-STAT signaling pathway genes (*JAK1, JAK2, STAT1, IRF1*, and* NOS2*) were successfully genotyped. There were no significant deviations of HWE in either the cases or controls. We did not observe significant differences in the genotype and allele distributions of any of the SNPs between the AAU patients and control subjects after Bonferroni correction (see Supplementary Table S1 in Supplementary Material available online at http://dx.doi.org/10.1155/2016/5896906). Further stratified analyses of gender, AS, and HLA-B27 status showed a boundary significant association of two SNPs (rs10975003 and rs10758669) of* JAK2* with AAU. In female AS-positive AAU patients, there was a decreased frequency of the TT genotype of rs10975003 compared to the female controls (OR = 0.55; *P* = 1.59 × 10^−3^; *P*_Bonferroni_ = 0.052, Table 3), whereas no significant differences were observed in the genotype and allele frequencies of the other ten SNPs (*P*_Bonferroni_ > 0.05, Table 3). Similarly, there were no significant differences in the genotype and allele frequencies of the SNPs between the male AAU patients and male controls (*P*_Bonferroni_ > 0.05, Supplementary Table S2).

In addition, an increased frequency of the AC genotype in rs10758669 was observed in HLA-B27-positive AAU patients compared to the healthy controls (OR = 1.44; *P* = 1.62 × 10^−3^; *P*_Bonferroni_ = 0.053, Table 4), whereas there were no significant differences in the genotype and allele frequencies of the other ten SNPs between the HLA-B27-positive AAU patients and the control subjects (*P*_Bonferroni_ > 0.05, Table 4).

However, an increased frequency of the rs10758669/AC genotype was observed in HLA-B27-positive AS-positive AAU patients compared to healthy controls (OR = 1.49; *P* = 2.56 × 10^−3^; *P*_Bonferroni_ = 0.084, Table 5), whereas no significant differences in the genotype and allele frequencies of the other 10 SNPs were observed between the HLA-B27-positive AS-positive AAU patients and healthy controls (*P*_Bonferroni_ > 0.05, Table 5). In addition, there were no significant differences in the genotype and allele frequencies of the tested SNPs between the AS-positive AAU patients and control subjects (*P*_Bonferroni_ > 0.05, Supplementary Table S3).

### 3.3. Logistic Regression Analysis of SNPs in AAU

We further investigated the SNPs rs10758669 and rs10975003 using additive, codominant, dominant, and recessive genetic models using a multivariate logistic regression model adjusted for age and gender. We observed that the frequency of the CA genotype of rs10758669 was significantly higher in AAU patients, which suggests that patients with the rs10758669 CA genotype have increased susceptibility to AAU (46.8% versus 40.6%, OR = 1.28, *P* = 0.02, Supplementary Table S4). An increased frequency of the AC genotype of rs10758669 was also observed in HLA-B27-positive AAU patients and AS-positive AAU patients compared to the healthy controls (49.5% versus 40.6%, OR = 1.43, *P* = 3.50 × 10^−3^ and 49.2% versus 40.6%, OR = 1.36, *P* = 0.02, Supplementary Table S4). For SNP rs10975003, the frequency of the heterozygous CT genotype was significantly higher in female AAU patients compared to the healthy controls (40.4% versus 33.6%, OR = 1.57, *P* = 0.01, Supplementary Table S5). A similar result was observed when we combined CT and CC to construct a dominant model (44.3% versus 38.0%, OR = 1.57, *P* = 9.10 × 10^−3^, Supplementary Table S5). However, none of the observed associations for the two SNPs retained statistical significance after Bonferroni correction (*P*_Bonferroni_ > 0.05). Furthermore, no significance associations were found between the other SNPs and AAU, even after stratification by gender, AS, and HLA-B27 status (data not shown).

## 4. Discussion

In this study, we first investigated whether genetic polymorphisms of JAK-STAT signaling pathway genes, including* JAK1, JAK2, STAT1, IRF1*, and* NOS2*, confer susceptibility to AAU with or without AS in a Chinese Han population. Our results suggest that none of these SNPs exhibit statistically different frequencies of genotypes and alleles between healthy controls and AAU patients. However, we observed a boundary significant association for two SNPs (rs10975003 and rs10758669) of* JAK2* by stratification analysis by gender and HLA-B27 status.

We highlighted two issues at the time of study design to obtain unbiased association results. First, we followed strict criteria for the diagnosis of AAU patients. Patients with AAU were diagnosed as previously described by Jabs et al. [36] and patients with AS were diagnosed with the modified New York Criteria [37]. In addition, the AAU patients and healthy controls were strictly matched by ethnicity and age to avoid a possible influence of population stratification. Furthermore, we only enrolled controls who had detailed histories and physical examinations and excluded controls with any autoimmune or immune-related diseases. Finally, 20% of the samples were randomly chosen and analyzed by direct sequencing, and the results of different genotyping methods were consistent.

AAU is defined as inflammation confined to the anterior segment of the eye that involves the iris and anterior part of the ciliary body. The HLA-B27 is considered to be strongly associated with both AAU and AS [5, 8, 10, 14]. Recent genetic studies have revealed that five candidate genes in the JAK-STAT signaling pathway were considered genetic predisposing factors for different autoimmune-mediated diseases [38–45]. SNPs (rs10758669 and 10975003) in* JAK2* are considered susceptibility factors for Crohn's disease (CD) in the German population and ulcerative colitis (UC) in the Korean population [38, 45]. One SNP (rs2070721) in* IRF1* and SNPs (rs2297518 and rs4795067) in* NOS2 *are also associated with autoimmune diseases, such as multiple sclerosis (MS) in Italy, AS in Europe, and psoriasis in Pakistan [39, 43, 44]. Four SNPs (rs6718902, rs10199181, rs2066802, and rs1547550) in the* STAT1* gene were observed to be associated with MS in Italy and IgA nephropathy (IgAN) in Korea [39, 40]. In addition, an association was found between SNPs (rs2780815, rs310241, rs3790532, rs310230, and rs310236) in* JAK1* and Behçet disease (BD) as well as Vogt-Koyanagi-Harada (VKH) syndrome [41, 42], two other common uveitis entities in China. There has been no report of associations between JAK-STAT signaling pathway genes and AAU, and thus we performed this case-control study to detect whether the five candidate genes were associated with AAU in a Chinese Han population. Our results showed that there were no significant associations between the genetic polymorphisms of the five candidate genes in the JAK-STAT signaling pathway and AAU. These results are not consistent with those observed for other autoimmune diseases reported in German, European, and some other Asian populations [38, 43, 45]. This discrepancy may be attributable to differences in the etiology and pathogenesis of AAU compared with BD, VKH syndrome, and autoimmune-mediated diseases.

Consistent with our results, a recent study also reported no significant association of JAK-STAT signaling pathway gene polymorphisms with rheumatoid arthritis stratified by the presence/absence of cardiovascular disease [46]. Conversely, an influence of NFKB1 signaling pathway polymorphisms on the development of cardiovascular events in patients with rheumatoid arthritis has been observed [47]. Furthermore, NFKB1 signaling pathway polymorphisms have been described to play a critical role in the development of many autoimmune and inflammatory diseases, and thus the evaluation of the potential relationships between* NFKB1* polymorphisms and the development of AAU could be a promising research line for the future.

There were several limitations of our study. We had a limited sample size to detect SNPs with weak effects while considering multiple corrections. Even for SNPs rs10758669 and rs10975003, we only had 70.0% power using a genetic power calculator [48]. In addition, our samples were restricted to the Han Chinese population, and all patients were enrolled from the ophthalmology department. Further studies with a larger sample size and other ethnic populations as well as patients enrolled from multiple sources are warranted to confirm our findings. Additionally, we only focused on eleven SNPs in the JAK-STAT pathway, and it is possible that other unknown SNPs might be associated with AAU risk.

In conclusion, this study reveals that genetic polymorphisms of JAK-STAT pathway genes, including* JAK1, JAK2, STAT1, IRF1*, and* NOS2*, may not be involved in susceptibility to AAU risk in the Han Chinese population.

## Supplementary Material

The allele and genotype frequencies of the candidate SNPs.

## Figures and Tables

**Table 1:** PCR primer sequences and restriction enzymes.

SNP	Primers	Restriction enzyme
rs310241	5′ AACCACCAGCTCAACATTCCTAG 3′ 5′ CAGCCAGGTCTCCCGTAGG 3′	BseDI
rs6718902	5′ CGGACAAAAGCATGCACTAGA 3′ 5′ CCACCACCATTAATAGGTGACTTTA 3′	DraI
rs2297518	5′ TGAGCTCTTTCAGCATGAAGATC 3′ 5′ CTTCCGTGGTGGGCTGTG 3′	TaqI
rs10758669	5′ TGATGTAGAGACAAGGACATGCTGAGGTAC 3′ 5′ GCCAAAAGACAAAGGCAAGGGG 3′	BanI
rs10975003	5′ GGCCAGTCAAGAAAAAACCAGTT 3′ 5′ TTCGGAGTCTTGTCTGAGCATGT 3′	HpaI
rs4795067	5′ GCACTCATTCATTCATGCAAACATA 3′ 5′ GGCAGAACTTGAACCCCAGCT 3′	NdeI
rs1547550	5′ CTTCTCTAGGAGGCCCAGCA 3′ 5′ TGGGCACCACGATATGAGAG 3′	BstMAI
rs2066802	5′ ATTCCTGGAGCAGGTTCACAAG 3′ 5′ AAACATGGCCCCAAGTCACT 3′	HindIII

**Table 2:** Clinical characteristics of the investigated subjects.

Clinical features	Total	Percentage
AAU patients	929	100
Mean age ± SD (years)	39.8 ± 12.3	
AAU with AS	443	47.7
AAU without AS	486	52.3
AAU male	569	61.2
AAU female	360	38.8
AAU with AS (male)	326 (443 tested)	73.6
AAU with AS (female)	117 (443 tested)	26.4
AAU without AS (male)	243 (486 tested)	50
AAU without AS (female)	243 (486 tested)	50
HLA-B27^+^ AAU	546 (792 tested)	68.9
HLA-B27^+^AAU AS^+^	348 (428 tested)	81.3
HLA-B27^+^AAU AS^−^	198 (364 tested)	54.4
Control	714	100
Mean age ± SD (years)	39.5 ± 10.8	
Male	428	59.9
Female	286	40.1

**Table 3:** Allele and genotype frequencies in female AAU patients and female controls.

Gene	SNP	Allele and genotype	AAU^+^AS^+^ (female)	AAU^+^AS^−^ (female)	Controls (female)	*P* (AS^+^)	Pc (AS^+^)	OR (95% CI)	*P* (AS^−^)	Pc (AS^−^)	OR (95% CI)
JAK1	rs310241	C	80 (33.3%)	138 (29.6%)	156 (28.7%)	0.19	NS	1.24 (0.90–1.72)	0.74	NS	1.05 (0.80–1.37)
		CC	12 (10.0%)	14 (6.0%)	17 (6.2%)	0.19	NS	1.67 (0.77–3.61)	0.91	NS	0.96 (0.46–2.00)
		CT	56 (46.7%)	110 (47.2%)	122 (44.9%)	0.74	NS	1.08 (0.70–1.66)	0.60	NS	1.10 (0.77–1.56)
		TT	52 (43.3%)	109 (46.8%)	133 (48.9%)	0.31	NS	0.80 (0.52–1.23)	0.64	NS	0.92 (0.65–1.30)
	rs2780815	G	159 (78.7%)	400 (87.0%)	495 (85.3%)	0.03	NS	0.64 (0.42–0.96)	0.45	NS	1.15 (0.80–1.63)
		GG	63 (62.4%)	175 (76.1%)	212 (73.1%)	0.04	NS	0.61 (0.38–0.99)	0.44	NS	1.17 (0.79–1.75)
		GT	33 (32.6%)	50 (21.7%)	71 (24.5%)	0.11	NS	1.50 (0.91–2.45)	0.46	NS	0.86 (0.57–1.29)
		TT	5 (5.0%)	5 (2.2%)	7 (2.4%)	0.20	NS	2.11 (0.65–6.80)	0.86	NS	0.90 (0.28–2.87)

JAK2	rs10758669	A	170 (73.9%)	328 (67.5%)	399 (72.3%)	0.64	NS	1.09 (0.77–1.54)	0.10	NS	0.80 (0.61–1.04)
		AA	59 (51.3%)	104 (42.8%)	144 (52.2%)	0.88	NS	0.97 (0.63–1.49)	0.03	NS	0.69 (0.49–1.00)
		AC	52 (45.2%)	120 (49.4%)	111 (40.2%)	0.36	NS	1.23 (0.79–1.90)	0.04	NS	1.45 (1.02–2.05)
		CC	4 (3.5%)	19 (7.8%)	21 (7.6%)	0.13	NS	0.44 (0.15–1.30)	0.93	NS	1.03 (0.54–1.97)
	rs10975003	C	44 (20.6%)	117 (25.8%)	93 (17.9%)	0.40	NS	1.19 (0.80–1.77)	2.83 × 10^−3^	0.09	1.59 (1.17–2.17)
		CC	2 (1.9%)	11 (4.8%)	8 (3.1%)	0.52	NS	0.60 (0.13–2.87)	0.32	NS	1.60 (0.63–4.06)
		CT	40 (37.4%)	95 (41.9%)	77 (29.6%)	0.15	NS	1.42 (0.88–2.28)	4.83 × 10^−3^	NS	1.71 (1.18–2.49)
		TT	65 (60.7%)	121 (53.3%)	175 (67.3%)	0.23	NS	0.75 (0.47–1.20)	1.59 × 10^−3^	0.052	0.55 (0.38–0.80)

STAT1	rs1547550	C	205 (86.9%)	411 (88.6%)	423 (87.0%)	0.95	NS	0.99 (0.62–1.56)	0.47	NS	1.16 (0.78–1.71)
		CC	88 (74.6%)	182 (78.4%)	185 (76.1%)	0.75	NS	0.92 (0.55–1.53)	0.55	NS	1.14 (0.74–1.75)
		CG	29 (24.6%)	47 (20.3%)	53 (21.8%)	0.56	NS	1.17 (0.70–1.96)	0.68	NS	0.91 (0.59–1.42)
		GG	1 (0.8%)	3 (1.3%)	5 (2.1%)	0.40	NS	0.41 (0.05–3.52)	0.56	NS	0.65 (0.15–2.76)
	rs2066802	C	46 (20.4%)	81 (17.4%)	113 (21.2%)	0.80	NS	0.95 (0.65–1.40)	0.13	NS	0.78 (0.57–1.08)
		CC	1 (0.9%)	6 (2.6%)	8 (3.0%)	0.22	NS	0.29 (0.04–2.34)	0.78	NS	0.86 (0.29–2.50)
		CT	44 (38.9%)	69 (29.6%)	97 (36.3%)	0.63	NS	1.12 (0.71–1.76)	0.11	NS	0.74 (0.51–1.07)
		TT	68 (60.2%)	158 (67.8%)	162 (60.7%)	0.93	NS	0.98 (0.63–1.54)	0.10	NS	1.37 (0.95–1.97)
	rs6718902	C	132 (60.0%)	259 (54.2%)	308 (53.8%)	0.12	NS	1.29 (0.94–1.76)	0.91	NS	1.01 (0.79–1.29)
		CC	36 (32.7%)	72 (30.1%)	82 (28.7%)	0.43	NS	1.21 (0.75–1.94)	0.72	NS	1.07 (0.74–1.56)
		CT	60 (54.5%)	115 (48.1%)	144 (50.3%)	0.45	NS	1.18 (0.76–1.84)	0.61	NS	0.92 (0.65–1.29)
		TT	14 (12.7%)	52 (21.8%)	60 (21.0%)	0.06	NS	0.55 (0.29–1.03)	0.83	NS	1.05 (0.69–1.59)
	rs10199181	A	51 (24.8%)	117 (26.1%)	148 (25.2%)	0.91	NS	0.98 (0.68–1.41)	0.73	NS	1.05 (0.79–1.39)
		AA	6 (5.8%)	20 (8.9%)	19 (6.5%)	0.82	NS	0.90 (0.35–2.31)	0.29	NS	1.42 (0.74–2.73)
		AT	39 (37.9%)	77 (34.4%)	110 (37.4%)	0.94	NS	1.02 (0.64–1.62)	0.48	NS	0.88 (0.61–1.26)
		TT	58 (56.3%)	127 (56.7%)	165 (56.1%)	0.97	NS	1.01 (0.64–1.58)	0.90	NS	1.02 (0.72–1.45)

IRF1	rs2070721	A	86 (40.6%)	154 (33.9%)	216 (34.3%)	0.10	NS	1.31 (0.95–1.80)	0.90	NS	0.98 (0.76–1.27)
		AA	15 (14.2%)	26 (11.5%)	29 (9.2%)	0.15	NS	1.63 (0.84–3.17)	0.39	NS	1.28 (0.73–2.23)
		AC	56 (52.8%)	102 (44.9%)	158 (50.2%)	0.63	NS	1.11 (0.72–1.73)	0.23	NS	0.81 (0.58–1.14)
		CC	35 (33.0%)	99 (43.6%)	128 (40.6%)	0.16	NS	0.72 (0.45–1.14)	0.49	NS	1.13 (0.80–1.60)

NOS2	rs2297518	A	52 (20.5%)	69 (15.2%)	74 (15.7%)	0.10	NS	1.39 (0.94–2.05)	0.84	NS	0.96 (0.68–1.38)
		AA	3 (2.4%)	6 (2.6%)	2 (0.8%)	0.24	NS	2.83 (0.47–17.17)	0.14	NS	3.18 (0.63–15.90)
		AG	46 (36.2%)	57 (25.2%)	70 (29.7%)	0.20	NS	1.35 (0.85–2.13)	0.27	NS	0.80 (0.53–1.20)
		GG	78 (61.4%)	164 (72.2%)	164 (69.5%)	0.12	NS	0.70 (0.45–1.10)	0.51	NS	1.14 (0.77–1.71)
	rs4795067	A	173 (73.9%)	369 (75.9%)	439 (77.8%)	0.24	NS	0.81 (0.57–1.15)	0.46	NS	0.90 (0.67–1.20)
		AA	62 (53.0%)	145 (59.7%)	169 (59.9%)	0.20	NS	0.75 (0.49–1.16)	0.95	NS	0.99 (0.70–1.40)
		AG	49 (41.9%)	79 (32.5%)	101 (35.8%)	0.26	NS	1.29 (0.83–2.01)	0.43	NS	0.86 (0.60–1.24)
		GG	6 (5.1%)	19 (7.8%)	12 (4.3%)	0.70	NS	1.22 (0.45–3.32)	0.08	NS	1.91 (0.91–4.02)

OR = odds ratio; 95% CI = 95% confidence interval.

Pc = *P* value adjusted by Bonferroni correction.

**Table 4:** Allele and genotype frequencies of SNPs in patients with AAU versus control subjects stratified by HLA-B27 status.

Gene	SNP	Allele and genotype	AAU HLA-B27	Control	*P*	Pc	OR (95% CI)
JAK1	rs310241	C	324 (31.0%)	392 (27.5%)	0.06	NS	1.18 (1.00–1.41)
		CC	41 (7.9%)	45 (6.3%)	0.29	NS	1.27 (0.82–1.96)
		CT	242 (46.4%)	302 (42.4%)	0.16	NS	1.18 (0.94–1.48)
		TT	239 (45.7%)	366 (51.3%)	0.05	NS	0.80 (0.64–1.00)
	rs2780815	G	878 (86.2%)	1250 (87.5%)	0.35	NS	0.89 (0.70–1.13)
		GG	381 (74.8%)	548 (76.7%)	0.44	NS	0.90 (0.69–1.18)
		GT	116 (22.8%)	154 (21.6%)	0.61	NS	1.07 (0.82–1.41)
		TT	12 (2.4%)	12 (1.7%)	0.40	NS	1.41 (0.63–3.17)

JAK2	rs10758669	A	705 (65.9%)	980 (69.0%)	0.10	NS	0.87 (0.73–1.03)
		AA	220 (41.1%)	346 (48.7%)	0.08	NS	0.74 (0.59–0.92)
		AC	265 (49.5%)	288 (40.6%)	1.62 × 10^−3^	0.053	1.44 (1.15–1.80)
		CC	50 (9.4%)	76 (10.7%)	0.43	NS	0.86 (0.59–1.25)
	rs10975003	C	251 (24.5%)	272 (21.2%)	0.05	NS	1.21 (1.00–1.47)
		CC	26 (5.1%)	28 (4.4%)	0.56	NS	1.18 (0.68–2.03)
		CT	199 (38.8%)	216 (33.5%)	0.06	NS	1.26 (0.99–1.60)
		TT	287 (56.1%)	399 (62.1%)	0.03	NS	0.77 (0.61–0.98)

STAT1	rs1547550	C	937 (87.2%)	1254 (87.8%)	0.67	NS	0.95 (0.75–1.21)
		CC	407 (75.8%)	555 (77.7%)	0.42	NS	0.90 (0.69–1.17)
		CG	123 (22.9%)	144 (20.2%)	0.24	NS	1.18 (0.90–1.54)
		GG	7 (1.3%)	15 (2.1%)	0.29	NS	0.62 (0.25–1.52)
	rs2066802	C	190 (19.3%)	288 (20.7%)	0.39	NS	0.91 (0.74–1.12)
		CC	15 (3.0%)	27 (3.9%)	0.44	NS	0.78 (0.41–1.48)
		CT	160 (32.5%)	234 (33.7%)	0.66	NS	0.95 (0.74–1.21)
		TT	318 (64.5%)	434 (62.4%)	0.47	NS	1.09 (0.86–1.39)
	rs6718902	C	584 (56.5%)	778 (54.5%)	0.33	NS	1.08 (0.92–1.27)
		CC	163 (31.5%)	209 (29.3%)	0.40	NS	1.11 (0.87–1.42)
		CT	258 (49.9%)	360 (50.4%)	0.86	NS	0.98 (0.78–1.23)
		TT	96 (18.6%)	145 (20.3%)	0.45	NS	0.90 (0.67–1.19)
	rs10199181	A	270 (27.2%)	397 (27.8%)	0.75	NS	0.97 (0.81–1.16)
		AA	43 (8.7%)	55 (7.7%)	0.55	NS	1.14 (0.75–1.73)
		AT	184 (37.1%)	287 (40.2%)	0.28	NS	0.88 (0.69–1.11)
		TT	269 (54.2%)	372 (52.1%)	0.47	NS	1.09 (0.87–1.37)

IRF1	rs2070721	A	356 (35.7%)	487 (34.1%)	0.40	NS	1.08(0.91–1.27)
		AA	61 (12.2%)	74 (10.4%)	0.30	NS	1.21 (0.84–1.73)
		AC	234 (47.0%)	339 (47.4%)	0.87	NS	0.98 (0.78–1.23)
		CC	203 (40.8%)	301 (42.2%)	0.63	NS	0.94 (0.75–1.19)

NOS2	rs2297518	A	194 (18.1%)	209 (16.2%)	0.21	NS	1.15 (0.93–1.42)
		AA	12 (2.3%)	12 (1.9%)	0.64	NS	1.21 (0.54–2.72)
		AG	170 (31.7%)	185 (28.6%)	0.24	NS	1.16 (0.90–1.49)
		GG	354 (66.0%)	450 (69.5%)	0.20	NS	0.85 (0.67–1.09)
	rs4795067	A	797 (74.3%)	1008 (76.1%)	0.35	NS	0.92 (0.76–1.10)
		AA	293 (54.6%)	382 (57.7%)	0.33	NS	0.89 (0.71–1.23)
		AG	211 (39.4%)	244 (36.9%)	0.37	NS	1.11 (0.88–1.41)
		GG	32 (6.0%)	36 (5.4%)	0.69	NS	1.10 (0.68–1.80)

OR = odds ratio; 95% CI = 95% confidence interval.

Pc = *P* value adjusted by Bonferroni correction.

**Table 5:** Allele and genotype frequencies of SNPs in patients with AAU versus control subjects stratified by AS and HLA-B27 status.

Gene	SNP	Allele and genotype	AAU^+^AS^+^ HLA-B27	AAU^+^AS^−^ HLA-B27	Control	*P* (AS^+^)	Pc (AS^+^)	OR (95% CI)	*P* (AS^−^)	Pc (AS^−^)	OR (95% CI)
JAK1	rs310241	C	205 (31.6%)	119 (29.9%)	392 (27.5%)	0.05	NS	1.22 (1.00–1.49)	0.32	NS	1.13 (0.88–1.45)
		CC	30 (9.3%)	11 (5.5%)	45 (6.3%)	0.09	NS	1.52 (0.94–2.45)	0.70	NS	0.87 (0.44–1.72)
		CT	145 (44.7%)	97 (48.7%)	302 (42.4%)	0.47	NS	1.10 (0.85–1.44)	0.10	NS	1.31 (0.95–1.79)
		TT	149 (46.0%)	90 (45.7%)	366 (51.3%)	0.11	NS	0.81 (0.62–1.05)	0.14	NS	0.79 (0.58–1.08)
	rs2780815	G	543 (84.3%)	335 (89.6%)	1250 (87.5%)	0.05	NS	0.77 (0.59–1.00)	0.28	NS	1.22 (0.85–1.77)
		GG	230 (71.4%)	151 (80.7%)	548 (76.7%)	0.07	NS	0.76 (0.56–1.02)	0.24	NS	1.27 (0.85–1.90)
		GT	83 (25.8%)	33 (17.6%)	154 (21.6%)	0.14	NS	1.26 (0.93–1.72)	0.23	NS	0.78 (0.51–1.18)
		TT	9 (2.8%)	3 (1.7%)	12 (1.7%)	0.24	NS	1.68 (0.70–4.03)	0.94	NS	0.95 (0.27–3.42)

JAK2	rs10758669	A	449 (66.2%)	256 (65.3%)	980 (69.0%)	0.20	NS	0.88 (0.72–1.70)	0.16	NS	0.85 (0.67–1.07)
		AA	139 (41.0%)	81 (41.3%)	346 (48.7%)	0.02	NS	0.73 (0.56–0.95)	0.07	NS	0.74 (0.54–1.02)
		AC	171 (50.4%)	94 (48.0%)	288 (40.6%)	2.56 × 10^−3^	0.084	1.49 (1.15–1.94)	0.06	NS	1.35 (0.98–1.86)
		CC	29 (8.6%)	21 (10.7%)	76 (10.7%)	0.28	NS	0.78 (0.50–1.22)	1.00	NS	1.00 (0.60–1.67)
	rs10975003	C	165 (25.7%)	86 (22.5%)	272 (21.2%)	0.03	NS	1.29 (1.03–1.61)	0.57	NS	1.08 (0.82–1.43)
		CC	18 (5.6%)	8 (4.2%)	28 (4.4%)	0.39	NS	1.31 (0.71–2.40)	0.92	NS	0.96 (0.43–2.14)
		CT	129 (40.2%)	70 (36.6%)	216 (33.5%)	0.04	NS	1.33 (1.01–1.75)	0.44	NS	1.14 (0.82–1.60)
		TT	174 (54.2%)	113 (59.2%)	399 (62.1%)	0.01	NS	0.72 (0.55–0.95)	0.47	NS	0.89 (0.64–1.23)

STAT1	rs1547550	C	608 (88.1%)	329 (85.7%)	1254 (87.8%)	0.84	NS	1.03 (0.78–1.36)	0.26	NS	0.83 (0.60–1.15)
		CC	264 (76.5%)	143 (74.5%)	555 (77.7%)	0.66	NS	0.93 (0.69–1.27)	0.34	NS	0.84 (0.58–1.21)
		CG	80 (23.2%)	43 (22.4%)	144 (20.2%)	0.26	NS	1.20 (0.88–1.63)	0.50	NS	1.14 (0.78–1.68)
		GG	1 (0.3%)	6 (3.1%)	15 (2.1%)	0.02	NS	0.14 (0.02–103)	0.40	NS	1.50 (0.58–3.93)
	rs2066802	C	130 (20.7%)	60 (16.8%)	288 (20.7%)	0.99	NS	1.00 (0.79–1.26)	0.09	NS	0.77 (0.57–1.05)
		CC	11 (3.5%)	4 (2.2%)	27 (3.9%)	0.77	NS	0.90 (0.44–1.83)	0.29	NS	0.57 (0.20–1.64)
		CT	108 (34.4%)	52 (29.1%)	234 (33.7%)	0.82	NS	1.03 (0.78–1.37)	0.24	NS	0.81 (0.56–1.16)
		TT	195 (62.1%)	123 (68.7%)	434 (62.4%)	0.92	NS	0.99 (0.75–1.30)	0.12	NS	1.32 (0.93–1.88)
	rs6718902	C	368 (57.7%)	216 (54.5%)	778 (54.5%)	0.18	NS	1.14 (0.94–1.38)	0.98	NS	1.00 (0.80–1.25)
		CC	103 (32.3%)	60 (30.3%)	209 (29.3%)	0.33	NS	1.15 (0.87–1.53)	0.78	NS	1.05 (0.75–1.48)
		CT	162 (50.8%)	96 (48.5%)	360 (50.4%)	0.91	NS	1.02 (0.78–1.32)	0.63	NS	0.93 (0.68–1.27)
		TT	54 (16.9%)	42 (21.2%)	145 (20.3%)	0.20	NS	0.80 (0.57–1.13)	0.78	NS	1.06 (0.72–1.56)
	rs10199181	A	170 (26.9%)	100 (27.8%)	397 (27.8%)	0.67	NS	0.96 (0.77–1.18)	0.99	NS	1.00 (0.77–1.29)
		AA	25 (7.9%)	18 (10.0%)	55 (7.7%)	0.91	NS	1.03 (0.63–1.68)	0.32	NS	1.33 (0.76–2.33)
		AT	120 (38.0%)	64 (35.6%)	287 (40.2%)	0.50	NS	0.91 (0.69–1.20)	0.26	NS	0.82 (0.58–1.15)
		TT	171 (54.1%)	98 (54.4%)	372 (52.1%)	0.55	NS	1.08 (0.83–1.41)	0.57	NS	1.10 (0.79–1.53)

IRF1	rs2070721	A	221 (34.9%)	135 (37.3%)	487 (34.1%)	0.73	NS	1.03 (0.85–1.26)	0.25	NS	1.15 (0.90-1.46)
		AA	37 (11.7%)	24 (13.3%)	74 (10.4%)	0.53	NS	1.14 (0.75–1.74)	0.26	NS	1.32 (0.81–2.16)
		AC	147 (46.4%)	87 (48.1%)	339 (47.4%)	0.74	NS	0.96 (0.73–1.25)	0.89	NS	1.02 (0.74–1.42)
		CC	133 (42.0%)	70 (38.6%)	301 (42.2%)	0.95	NS	0.99 (0.76–1.30)	0.40	NS	0.97 (0.62–1.21)

NOS2	rs2297518	A	130 (19.0%)	64 (16.5%)	209 (16.2%)	0.11	NS	1.22 (0.96–1.55)	0.87	NS	1.03 (0.76–1.39)
		AA	7 (2.0%)	5 (2.6%)	12 (1.9%)	0.83	NS	1.11 (0.43–2.84)	0.53	NS	1.40 (0.49–4.02)
		AG	116 (33.9%)	54 (27.8%)	185 (28.6%)	0.08	NS	1.28 (0.97–1.70)	0.84	NS	0.96 (0.67–1.38)
		GG	219 (64.1%)	135 (69.6%)	450 (69.5%)	0.07	NS	0.78 (0.59–1.03)	0.99	NS	1.00 (0.71–1.42)
	rs4795067	A	512 (73.6%)	295 (74.5%)	1008 (76.1%)	0.20	NS	0.87 (0.71–1.08)	0.61	NS	0.92 (0.71–1.19)
		AA	184 (52.9%)	109 (55.1%)	382 (57.7%)	0.14	NS	0.82 (0.63–1.07)	0.51	NS	0.90 (0.65–1.24)
		AG	144 (41.4%)	77 (38.8%)	244 (36.9%)	0.16	NS	1.21 (0.93–1.58)	0.60	NS	1.09 (0.79–1.51)
		GG	20 (5.7%)	12 (6.1%)	36 (5.4%)	0.84	NS	1.06 (0.60–1.86)	0.74	NS	1.12 (0.57–2.20)

OR = odds ratio; 95% CI = 95% confidence interval.

Pc = *P* value adjusted by Bonferroni correction.

## References

[1] Hwang D.-K., Chou Y.-J., Pu C.-Y., Chou P. (2012). Epidemiology of uveitis among the Chinese population in Taiwan: A Population-Based Study. *Ophthalmology*.

[2] Nakao K., Ohba N. (1996). Prevalence of endogenous uveitis in Kagoshima Prefecture, Southwest Japan. *Nippon Ganka Gakkai Zasshi*.

[3] Gritz D. C., Wong I. G. (2004). Incidence and prevalence of uveitis in Northern California: the Northern California Epidemiology of Uveitis Study. *Ophthalmology*.

[4] Nussenblatt R. B., Gery I. (1996). Experimental autoimmune uveitis and its relationship to clinical ocular inflammatory disease. *Journal of Autoimmunity*.

[5] Yang P., Zhang Z., Zhou H. (2005). Clinical patterns and characteristics of uveitis in a tertiary center for uveitis in China. *Current Eye Research*.

[6] Gran J. T., Skomsvoll J. F. (1997). The outcome of ankylosing spondylitis: a study of 100 patients. *British Journal of Rheumatology*.

[7] Robertson L. P., Davis M. J. (2004). A longitudinal study of disease activity and functional status in a hospital cohort of patients with ankylosing spondylitis. *Rheumatology*.

[8] Chang J. H., McCluskey P. J., Wakefield D. (2005). Acute anterior uveitis and HLA-B27. *Survey of Ophthalmology*.

[9] Huhtinen M., Karma A. (2000). HLA-B27 typing in the categorisation of uveitis in a HLA-B27 rich population. *British Journal of Ophthalmology*.

[10] Torres S., Borges S., Artiles A. (2013). HLA-B27 and clinical features of acute anterior uveitis in Cuba. *Ocular Immunology and Inflammation*.

[11] Saari R., Lahti R., Saari K. M. (1982). Frequency of rheumatic diseases in patients with acute anterior uveitis. *Scandinavian Journal of Rheumatology*.

[12] Beckingsale A. B., Davies J., Gibson J. M., Ralph Rosenthal A. (1984). Acute anterior uveitis, ankylosing spondylitis, back pain, and HLA-B27. *British Journal of Ophthalmology*.

[13] Thomas G. P., Brown M. A. (2010). Genetics and genomics of ankylosing spondylitis. *Immunological Reviews*.

[14] Robinson P. C., Brown M. A. (2014). Genetics of ankylosing spondylitis. *Molecular Immunology*.

[15] Lopez-Larrea C., Sujirachato K., Mehra N. K. (1995). HLA-B27 subtypes in Asian patients with ankylosing spondylitis. Evidence for new associations. *Tissue Antigens*.

[16] Robinson P. C., Claushuis T. A. M., Cortes A. (2015). Genetic dissection of acute anterior uveitis reveals similarities and differences in associations observed with ankylosing spondylitis. *Arthritis and Rheumatology*.

[17] Li H., Hou S., Yu H. (2015). Association of genetic variations in TNFSF15 with acute anterior uveitis in Chinese Han. *Investigative Ophthalmology and Visual Science*.

[18] Xiang Q., Chen L., Fang J. (2013). TNF receptor-associated factor 5 gene confers genetic predisposition to acute anterior uveitis and pediatric uveitis. *Arthritis Research and Therapy*.

[19] Yu H., Liu Y., Zhang L. (2014). *FoxO1* gene confers genetic predisposition to acute anterior uveitis with ankylosing spondylitis. *Investigative Ophthalmology & Visual Science*.

[20] Martin T. M., Bye L., Modi N. (2009). Genotype analysis of polymorphisms in autoimmune susceptibility genes, CTLA-4 and PTPN22, in an Acute anterior uveitis cohort. *Molecular Vision*.

[21] Zhang Q., Qi J., Hou S. (2014). A functional variant of PTPN22 confers risk for Vogt-Koyanagi-Harada syndrome but not for ankylosing spondylitis. *PLoS ONE*.

[22] Zhao S.-S., Hu J.-W., Wang J., Lou X.-J., Zhou L.-L. (2011). Inverse correlation between CD4^+^CD25^ high^CD127^low/−^ regulatory T-cells and serum immunoglobulin A in patients with new-onset ankylosing spondylitis. *Journal of International Medical Research*.

[23] Zou W., Wu Z., Xiang X., Sun S., Zhang J. (2014). The expression and significance of T helper cell subsets and regulatory T cells CD4+CD25+ in peripheral blood of patients with human leukocyte antigen B27-positive acute anterior uveitis. *Graefe's Archive for Clinical and Experimental Ophthalmology*.

[24] Wang C., Liao Q., Hu Y., Zhong D. (2015). T lymphocyte subset imbalances in patients contribute to ankylosing spondylitis. *Experimental and Therapeutic Medicine*.

[25] Tedgui A., Mallat Z. (2006). Cytokines in atherosclerosis: pathogenic and regulatory pathways. *Physiological Reviews*.

[26] Murray P. J. (2007). The JAK-STAT signaling pathway: Input and output integration. *Journal of Immunology*.

[27] Mathur A. N., Chang H.-C., Zisoulis D. G. (2007). Stat3 and Stat4 direct development of IL-17-secreting Th cells. *Journal of Immunology*.

[28] Hansson G. K. (2001). Regulation of immune mechanisms in atherosclerosis. *Annals of the New York Academy of Sciences*.

[29] Shea-Donohue T., Fasano A., Smith A., Zhao A. (2010). Enteric pathogens and gut function: role of cytokines and STATs. *Gut Microbes*.

[30] Taniguchi T., Ogasawara K., Takaoka A., Tanaka N. (2001). IRF family of transcription factors as regulators of host defense. *Annual Review of Immunology*.

[31] Lohoff M., Ferrick D., Mittrücker H.-W. (1997). Interferon regulatory factor-1 is required for a T helper 1 immune response in vivo. *Immunity*.

[32] Taki S., Sato T., Ogasawara K. (1997). Multistage regulation of Th1-type immune responses by the transcription factor IRF-1. *Immunity*.

[33] Bogdan C. (2001). Nitric oxide and the immune response. *Nature Immunology*.

[34] Brahmachari S., Pahan K. (2010). Myelin basic protein priming reduces the expression of Foxp3 in T cells via nitric oxide. *The Journal of Immunology*.

[35] Lee S.-W., Choi H., Eun S.-Y., Fukuyama S., Croft M. (2011). Nitric oxide modulates TGF-*β*-directive signals to suppress Foxp3 + regulatory T cell differentiation and potentiate Th1 development. *Journal of Immunology*.

[36] Jabs D. A., Nussenblatt R. B., Rosenbaum J. T., Standardization of Uveitis Nomenclature Working G (2005). Standardization of uveitis nomenclature for reporting clinical data. Results of the First International Workshop. *American Journal of Ophthalmology*.

[37] Van Der Linden S., Valkenburg H. A., Cats A. (1984). Evaluation of diagnostic criteria for ankylosing spondylitis. A proposal for modification of the New York criteria. *Arthritis and Rheumatism*.

[38] Ellinghaus D., Ellinghaus E., Nair R. P. (2012). Combined analysis of genome-wide association studies for Crohn disease and psoriasis identifies seven shared susceptibility loci. *American Journal of Human Genetics*.

[39] Fortunato G., Calcagno G., Bresciamorra V. (2008). Multiple sclerosis and hepatitis C virus infection are associated with single nucleotide polymorphisms in interferon pathway genes. *Journal of Interferon and Cytokine Research*.

[40] Hahn W.-H., Suh J.-S., Cho S. H., Cho B.-S., Kim S.-D. (2010). Polymorphisms of signal transducers and activators of transcription 1 and 4 (STAT1 and STAT4) contribute to progression of childhood IgA nephropathy. *Cytokine*.

[41] Hou S., Qi J., Zhang Q. (2013). Genetic variants in the JAK1 gene confer higher risk of Behcet's disease with ocular involvement in Han Chinese. *Human Genetics*.

[42] Hu K., Hou S., Li F., Xiang Q., Kijlstra A., Yang P. (2013). JAK1, but not JAK2 and STAT3, confers susceptibility to Vogt-Koyanagi-Harada (VKH) syndrome in a Han Chinese population. *Investigative Ophthalmology and Visual Science*.

[43] Cortes A., Hadler J., Pointon J. P. (2013). Identification of multiple risk variants for ankylosing spondylitis through high-density genotyping of immune-related loci. *Nature Genetics*.

[44] Shaiq P. A., Stuart P. E., Latif A. (2013). Genetic associations of psoriasis in a Pakistani population. *British Journal of Dermatology*.

[45] Yang S.-K., Jung Y., Kim H., Hong M., Ye B. D., Song K. (2011). Association of FCGR2A, JAK2 or HNF4A variants with ulcerative colitis in Koreans. *Digestive and Liver Disease*.

[46] García-Bermúdez M., López-Mejías R., Genre F. (2015). Lack of association between *JAK3* gene polymorphisms and cardiovascular disease in Spanish patients with rheumatoid arthritis. *BioMed Research International*.

[47] López-Mejías R., García-Bermúdez M., González-Juanatey C. (2012). NFKB1-94ATTG ins/del polymorphism (rs28362491) is associated with cardiovascular disease in patients with rheumatoid arthritis. *Atherosclerosis*.

[48] Park H. J., Kim J. W., Cho B.-S., Chung J.-H. (2014). Association of BH3 interacting domain death agonist (BID) gene polymorphisms with proteinuria of immunoglobulin A nephropathy. *Scandinavian Journal of Clinical and Laboratory Investigation*.

