# Supplementary material for: Lack of Association between Genetic Polymorphisms of JAK-STAT Signaling Pathway Genes and Acute Anterior Uveitis in Han Chinese
Source: Biomed Res Int. 2016 Nov 14;2016:5896906. doi: 10.1155/2016/5896906 (PMC5124643; doi:10.1155/2016/5896906)
Supplement: Supplementary file 1 — The allele and genotype frequencies of the candidate SNPs. [file 5896906.f1.pdf]

Supplementary Information

Table S1. Allele and genotype frequencies in AAU

Gene	SNP	Allele and Genotype	AAU	Controls	P	Pc	OR(95%CI)
JAK1	rs310241	C	555(30.6%)	392(27.5%)	0.06	NS	1.16(1.00-1.35)
		CC	74(8.2%)	45(6.3%)	0.16	NS	1.32(0.90-1.93)
		CT	407(44.8%)	302(42.4%)	0.32	NS	1.11(0.91-1.35)
		TT	427(47.0%)	366(51.3%)	0.09	NS	0.84(0.70-1.02)
	rs2780815	G	1478(86.6%)	1250(87.5%)	0.46	NS	0.92(0.75-1.14)
		GG	642(75.3%)	548(76.7%)	0.49	NS	0.92(0.73-1.16)
		GT	194(22.7%)	154(21.6%)	0.58	NS	1.07(0.84-1.36)
		TT	17(2.0%)	12(1.7%)	0.65	NS	1.19(0.56-2.51)
JAK2	rs10758669	A	1242(67.3%)	980(69.0%)	0.30	NS	0.92(0.80-1.07)
		AA	405(43.9%)	346(48.7%)	0.05	NS	0.82(0.68-1.00)
		AC	432(46.8%)	288(40.6%)	0.01	NS	1.29(1.06-1.57)
		CC	86(9.3%)	76(10.7%)	0.35	NS	0.86(0.62-1.18)
	rs10975003	C	406(23.7%)	272(21.2%)	0.10	NS	1.16(0.97-1.38)

		CC	42(4.9%)	28(4.4%)	0.62	NS	1.13(0.70-1.85)
		CT	322(37.6%)	216(33.6%)	0.11	NS	1.19(0.96-1.48)
		TT	492(57.5%)	399(62.1%)	0.07	NS	0.83(0.67-1.02)
STAT1	rs1547550	C	1601(87.6%)	1254(87.8%)	0.84	NS	0.98(0.80-1.21)
		CC	697(76.3%)	555(77.7%)	0.48	NS	0.92(0.73-1.16)
		CG	207(22.6%)	144(20.2%)	0.23	NS	1.16(0.91-1.48)
		GG	10(1.1%)	15(2.1%)	0.10	NS	0.52(0.23-1.15)
	rs2066802	C	346(19.7%)	288(20.7%)	0.47	NS	0.94(0.79-1.12)
		CC	29(3.3%)	27(3.9%)	0.87	NS	0.95(0.56-1.63)
		CT	288(32.8%)	234(33.7%)	0.75	NS	0.96(0.78-1.19)
		TT	562(63.9%)	434(62.4%)	0.54	NS	1.07(0.87-1.31)
	rs6718902	C	1017(56.2%)	778(54.5%)	0.32	NS	1.07(0.93-1.24)
		CC	289(32.0%)	209(29.3%)	0.24	NS	1.14(0.94-1.27)
		CT	439(48.5%)	360(50.4%)	0.46	NS	0.93(0.76-1.13)
		TT	176(19.5%)	145(20.3%)	0.67	NS	0.95(0.74-1.21)
	rs10199181	A	453(27.0%)	397(27.8%)	0.62	NS	0.96(0.82-1.13)
		AA	72(8.6%)	55(7.7%)	0.53	NS	1.13(0.78-1.62)

		AT	309(36.8%)	287(40.2%)	0.17	NS	0.87(0.71-1.07)
		TT	458(54.6%)	372(52.1%)	0.33	NS	1.11(0.91-1.35)
IRF1	rs2070721	A	590(34.9%)	487(34.1%)	0.65	NS	1.04(0.89-1.20)
		AA	98(11.6%)	74(10.4%)	0.44	NS	1.13(0.82-1.56)
		AC	394(46.6%)	339(47.4%)	0.72	NS	0.96(0.79-1.18)
		CC	354(41.8%)	301(42.2%)	0.90	NS	0.99(0.81-1.21)
NOS2	rs2297518	A	317(17.6%)	209(16.2%)	0.30	NS	1.11(0.91-1.34)
		AA	22(2.4%)	12(1.9%)	0.44	NS	1.32(0.65-2.69)
		AG	273(30.3%)	185(28.6%)	0.48	NS	1.08(0.87-1.35)
		GG	607(67.3%)	450(69.5%)	0.35	NS	0.90(0.73-1.12)
	rs4795067	A	1377(74.1%)	1008(76.1%)	0.19	NS	0.90(0.76-1.06)
		AA	514(55.3%)	382(57.7%)	0.35	NS	0.91(0.74-1.11)
		AG	349(37.6%)	244(36.9%)	0.77	NS	1.03(0.84-1.27)
		GG	66(7.1%)	36(5.4%)	0.18	NS	1.33(0.88-2.02)

OR = odds ratio; 95%CI = 95% confidence interval;

Pc = P value adjusted by Bonferroni correction.

Table S2. Allele and genotype frequencies in male AAU patients and male controls

Gene	SNP	Allele and Genotype	AAU^+AS^+ (Male)	AAU^+AS^- (Male)	Controls (Male)	P (AS^+)	Pc (AS^+)	OR (95%CI)	P (AS^-)	Pc (AS^-)	OR (95%CI)
JAK1	rs310241	C	193(29.9%)	144(31.0%)	236(26.8%)	0.18	NS	1.17(0.93-1.46)	0.10	NS	1.23(0.96-1.58)
		CC	29(9.0%)	19(8.2%)	28(6.4%)	0.17	NS	1.46(0.85-2.50)	0.37	NS	1.32(0.72-2.41)
		CT	135(41.8%)	106(45.7%)	180(40.8%)	0.79	NS	1.04(0.78-1.39)	0.22	NS	1.22(0.89-1.68)
		TT	159(49.2%)	107(46.1%)	233(52.8%)	0.32	NS	0.87(0.65-1.15)	0.09	NS	0.76(0.56-1.05)
	rs2780815	G	524(87.0%)	395(89.4%)	755(89.0%)	0.25	NS	0.83(0.60-1.14)	0.86	NS	1.04(0.71-1.50)
		GG	228(75.7%)	176(79.6%)	336(79.2%)	0.26	NS	0.82(0.58-1.16)	0.91	NS	1.02(0.69-1.53)
		GT	68(22.6%)	43(19.5%)	83(19.6%)	0.32	NS	1.20(0.84-1.72)	0.97	NS	0.99(0.66-1.50)
		TT	5(1.7%)	2(0.9%)	5(1.2%)	0.58	NS	1.42(0.41-4.93)	0.75	NS	0.77(0.15-3.98)
JAK2	rs10758669	A	415(64.4%)	329(67.7%)	537(67.1%)	0.29	NS	0.89(0.71-1.10)	0.83	NS	1.21(0.81-1.31)
		AA	126(39.1%)	116(47.7%)	202(46.5%)	0.04	NS	0.74(0.55-0.99)	0.77	NS	1.05(0.77-1.44)
		AC	163(50.6%)	97(39.9%)	177(40.8%)	7.17×10^{-3}	NS	1.49(1.11-1.99)	0.83	NS	0.97(0.70-1.33)
		CC	33(10.2%)	30(12.3%)	55(12.7%)	0.30	NS	0.79(0.50-1.24)	0.90	NS	0.97(0.60-1.56)
	rs10975003	C	155(26.4%)	90(19.7%)	179(23.4%)	0.21	NS	1.17(0.92-1.51)	0.14	NS	0.81(0.61-1.07)
		CC	20(6.8%)	9(3.9%)	20(5.2%)	0.39	NS	1.33(0.70-2.51)	0.47	NS	0.75(0.33-1.67)

		CT	115(39.1%)	72(31.6%)	139(36.3%)	0.45	NS	1.13(0.82-1.54)	0.24	NS	0.81(0.57-1.15)
		TT	159(54.1%)	147(64.5%)	224(58.5%)	0.25	NS	0.84(0.62-1.14)	0.14	NS	1.29(0.92-1.81)
STAT1	rs1547550	C	571(87.8%)	414(86.6%)	659(87.9%)	0.99	NS	1.00(0.72-1.38)	0.52	NS	0.89(0.63-1.26)
		CC	246(75.7%)	181(75.7%)	292(77.9%)	0.50	NS	0.89(0.62-1.26)	0.54	NS	0.88(0.61-1.30)
		CG	79(24.3%)	52(21.8%)	75(20.0%)	0.17	NS	1.29(0.90-1.84)	0.60	NS	1.11(0.75-1.66)
		GG	0(0.0%)	6(2.5%)	8(2.1%)	-	-	-	0.76	NS	1.18(0.41-3.45)
	rs2066802	C	127(21.0%)	92(20.0%)	175(20.4%)	0.81	NS	1.03(0.80-1.33)	0.85	NS	0.97(0.73-1.29)
		CC	14(4.6%)	8(3.5%)	19(4.4%)	0.91	NS	1.04(0.51-2.11)	0.55	NS	0.78(0.33-1.80)
		CT	99(32.7%)	76(33.0%)	137(32.0%)	0.85	NS	1.03(0.75-1.41)	0.79	NS	1.05(0.75-1.48)
		TT	190(62.7%)	146(63.5%)	272(63.6%)	0.82	NS	0.96(0.71-1.31)	0.99	NS	1.00(0.72-1.39)
	rs6718902	C	349(55.6%)	277(57.5%)	470(54.9%)	0.80	NS	1.03(0.84-1.26)	0.37	NS	1.11(0.89-1.39)
		CC	102(32.5%)	79(32.8%)	127(29.7%)	0.41	NS	1.14(0.83-1.56)	0.40	NS	1.16(0.82-1.62)
		CT	145(46.2%)	119(49.4%)	216(50.4%)	0.25	NS	0.84(0.63-1.13)	0.79	NS	0.96(0.70-1.31)
		TT	67(21.3%)	43(17.8%)	85(19.9%)	0.62	NS	1.10(0.76-1.57)	0.52	NS	0.88(0.58-1.32)
	rs10199181	A	165(27.9%)	120(27.8%)	249(29.6%)	0.47	NS	0.92(0.73-1.16)	0.49	NS	0.91(0.71-1.18)
		AA	30(10.1%)	16(7.4%)	36(8.6%)	0.48	NS	1.20(0.72-2.00)	0.61	NS	0.85(0.46-1.58)
		AT	105(35.5%)	88(40.7%)	177(42.1%)	0.07	NS	0.76(0.56-1.03)	0.73	NS	0.94(0.68-1.32)

		TT	161(54.4%)	112(51.9%)	207(49.3%)	0.18	NS	1.23(0.91-1.65)	0.54	NS	1.11(0.80-1.54)
IRF1	rs2070721	A	197(33.6%)	153(34.8%)	271(34.0%)	0.89	NS	0.99(0.79-1.23)	0.77	NS	1.04(0.81-1.32)
		AA	29(9.9%)	28(12.7%)	45(11.3%)	0.56	NS	0.86(0.53-1.42)	0.59	NS	1.15(0.69-1.90)
		AC	139(47.4%)	97(44.1%)	181(45.3%)	0.59	NS	1.09(0.80-1.47)	0.76	NS	0.95(0.68-1.32)
		CC	125(42.7%)	95(43.2%)	173(43.4%)	0.86	NS	0.97(0.72-1.32)	0.97	NS	0.99(0.71-1.38)
NOS2	rs2297518	A	114(18.1%)	82(17.6%)	133(16.3%)	0.36	NS	1.14(0.86-1.50)	0.54	NS	1.10(.81-1.49)
		AA	7(2.3%)	6(2.6%)	10(2.4%)	0.84	NS	0.91(0.34-2.41)	0.92	NS	1.06(0.38-2.94)
		AG	100(31.7%)	70(30.0%)	113(27.6%)	0.23	NS	1.22(0.88-1.68)	0.52	NS	1.13(0.79-1.60)
		GG	208(66.0%)	157(67.4%)	286(70.0%)	0.26	NS	0.84(0.61-1.15)	0.50	NS	0.89(0.63-1.26)
	rs4795067	A	476(73.0%)	359(73.9%)	569(74.9%)	0.43	NS	0.91(0.72-1.15)	0.69	NS	0.95(0.73-1.23)
		AA	171(52.5%)	136(56.0%)	213(56.1%)	0.34	NS	0.87(0.64-1.16)	0.98	NS	1.00(0.72-1.38)
		AG	134(41.1%)	87(35.8%)	143(37.6%)	0.35	NS	1.16(0.85-1.57)	0.64	NS	0.92(0.66-1.29)
		GG	21(6.4%)	20(8.2%)	24(6.3%)	0.95	NS	1.02(0.56-1.87)	0.36	NS	1.33(0.72-2.47)

OR = odds ratio; 95%CI = 95% confidence interval;

Pc = P value adjusted by Bonferroni correction.

Table S3. Allele and genotype frequencies in AAU stratified by AS status

Gene	SNP	Allele and Genotype	AAU^+AS^+	AAU^+AS^-	Controls	P (AAU^+AS^+)	Pc (AAU^+AS^+)	OR (95%CI)	P (AAU^+AS^-)	Pc (AAU^+AS^-)	OR (95%CI)
JAK1	rs310241	C	273(30.8%)	282(30.3%)	392(27.5%)	0.09	NS	1.18(0.98-1.41)	0.14	NS	1.15(0.96-1.38)
		CC	41(9.3%)	33(7.0%)	45(6.3%)	0.06	NS	1.51(0.97-2.35)	0.73	NS	1.13(0.71-1.81)
		CT	191(43.1%)	216(46.5%)	302(42.4%)	0.80	NS	1.03(0.81-1.31)	0.17	NS	1.18(0.93-1.49)
		TT	211(47.6%)	216(46.5%)	366(51.3%)	0.22	NS	0.86(0.68-1.09)	0.10	NS	0.82(0.65-1.04)
	rs2780815	G	683(85.0%)	795(88.1%)	1250(87.5%)	0.08	NS	0.80(0.63-1.03)	0.66	NS	1.06(0.82-1.37)
		GG	291(72.4%)	351(77.8%)	548(76.7%)	0.11	NS	0.79(0.60-1.05)	0.67	NS	1.06(0.80-1.41)
		GT	101(25.1%)	93(20.6%)	154(21.6%)	0.17	NS	1.22(0.92-1.63)	0.70	NS	0.95(0.71-1.26)
		TT	10(2.5%)	7(1.6%)	12(1.7%)	0.35	NS	1.49(0.64-3.49)	0.87	NS	0.92(0.36-2.36)
JAK2	rs10758669	A	585(66.9%)	657(67.6%)	980(69.0%)	0.30	NS	0.91(0.76-1.09)	0.47	NS	0.94(0.79-1.12)
		AA	185(42.3%)	220(45.3%)	346(48.7%)	0.04	NS	0.77(0.61-0.98)	0.24	NS	0.87(0.69-1.10)
		AC	215(49.2%)	217(44.7%)	288(40.6%)	4.21×10^{-3}	NS	1.42(1.12-1.80)	0.16	NS	1.18(0.94-1.49)
		CC	37(8.5%)	49(10.0%)	76(10.7%)	0.22	NS	0.77(0.51-1.17)	0.70	NS	0.93(0.64-1.36)
	rs10975003	C	199(24.8%)	207(22.7%)	272(21.2%)	0.05	NS	1.23(0.10-1.52)	0.37	NS	1.10(0.89-1.35)
		CC	22(5.5%)	20(4.4%)	28(4.4%)	0.41	NS	1.28(0.72-2.26)	0.97	NS	1.01(0.56-1.82)

		CT	155(38.7%)	167(36.7%)	216(33.5%)	0.10	NS	1.25(0.96-1.61)	0.28	NS	1.15(0.89-1.47)
		TT	224(55.8%)	268(58.9%)	399(62.1%)	0.05	NS	0.77(0.60-0.10)	0.29	NS	0.88(0.69-1.12)
STAT1	rs1547550	C	776(87.6%)	825(87.6%)	1254(87.8%)	0.87	NS	0.98(0.76-1.26)	0.86	NS	0.98(0.76-1.26)
		CC	334(75.4%)	363(77.1%)	555(77.7%)	0.36	NS	0.88(0.66-1.16)	0.79	NS	0.96(0.73-1.27)
		CG	108(24.4%)	99(21.0%)	144(20.2%)	0.09	NS	1.28(0.96-1.70)	0.72	NS	1.05(0.79-1.40)
		GG	1(0.2%)	9(1.9%)	15(2.1%)	0.01	NS	0.11(0.01-0.80)	0.82	NS	0.91(0.39-2.09)
	rs2066802	C	173(20.8%)	173(18.7%)	288(20.7%)	0.97	NS	1.00(0.81-1.24)	0.23	NS	0.88(0.71-1.08)
		CC	15(3.6%)	14(3.0%)	27(3.9%)	0.82	NS	0.93(0.49-1.76)	0.44	NS	0.77(0.40-1.49)
		CT	143(34.4%)	145(31.3%)	234(33.7%)	0.81	NS	1.03(0.80-1.33)	0.40	NS	0.90(0.70-1.16)
		TT	258(62.0%)	304(65.7%)	434(62.4%)	0.89	NS	0.98(0.76-1.26)	0.27	NS	1.15(0.90-1.47)
	rs6718902	C	481(56.7%)	536(55.8%)	778(54.5%)	0.30	NS	1.10(0.92-1.30)	0.52	NS	1.06(0.90-1.25)
		CC	138(32.5%)	151(31.5%)	209(29.3%)	0.25	NS	1.17(0.90-1.51)	0.42	NS	1.11(0.86-1.43)
		CT	205(48.3%)	234(48.7%)	360(50.4%)	0.50	NS	0.92(0.72-1.17)	0.57	NS	0.94(0.74-1.18)
		TT	81(19.2%)	95(19.8%)	145(20.3%)	0.62	NS	0.93(0.68-1.26)	0.83	NS	0.97(0.73-1.30)
	rs10199181	A	216(27.1%)	237(26.9%)	397(27.8%)	0.71	NS	0.96(0.80-1.17)	0.65	NS	0.96(0.79-1.16)
		AA	36(9.0%)	36(8.2%)	55(7.7%)	0.44	NS	1.19(0.77-1.84)	0.77	NS	1.07(0.69-1.66)
		AT	144(36.1%)	165(37.5%)	287(40.2%)	0.18	NS	0.84(0.65-1.08)	0.36	NS	0.89(0.70-1.14)

		TT	219(54.9%)	239(54.3%)	372(52.1%)	0.37	NS	1.12(0.88-1.43)	0.46	NS	1.09(0.86-1.39)
IRF1	rs2070721	A	283(35.5%)	307(34.3%)	487(34.1%)	0.52	NS	1.06(0.89-1.27)	0.91	NS	1.01(0.85-1.21)
		AA	44(11.0%)	54(12.1%)	74(10.4%)	0.73	NS	1.07(0.72-1.59)	0.36	NS	1.19(0.82-1.73)
		AC	195(48.9%)	199(44.5%)	339(47.4%)	0.66	NS	1.06(0.83-1.35)	0.33	NS	0.89(0.70-1.13)
		CC	160(40.1%)	194(43.4%)	301(42.2%)	0.50	NS	0.92(0.72-1.18)	0.68	NS	1.05(0.83-1.34)
NOS2	rs2297518	A	166(18.8%)	151(16.4%)	209(16.2%)	0.11	NS	1.20(0.96-1.50)	0.87	NS	1.02(0.81-1.28)
		AA	10(2.3%)	12(2.6%)	12(1.9%)	0.64	NS	1.22(0.53-2.86)	0.40	NS	1.42(0.63-3.19)
		AG	146(33.0%)	127(27.6%)	185(28.6%)	0.12	NS	1.23(0.95-1.60)	0.72	NS	0.95(0.73-1.24)
		GG	286(64.7%)	321(69.8%)	450(69.5%)	0.09	NS	0.80(0.62-1.04)	0.93	NS	1.01(0.78-1.31)
	rs4795067	A	649(73.3%)	728(74.9%)	1008(76.1%)	0.13	NS	0.86(0.71-1.04)	0.50	NS	0.94(0.77-1.13)
		AA	233(52.6%)	281(57.8%)	382(57.7%)	0.09	NS	0.81(0.64-1.04)	0.97	NS	1.01(0.79-1.27)
		AG	183(41.3%)	166(34.2%)	244(36.9%)	0.14	NS	1.21(0.94-1.54)	0.35	NS	0.89(0.70-1.14)
		GG	27(6.1%)	39(8.0%)	36(5.4%)	0.64	NS	1.13(0.68-1.89)	0.08	NS	1.52(0.95-2.43)

OR = odds ratio; 95%CI = 95% confidence interval;

Pc = P value adjusted by Bonferroni correction

Table S4. Multivariate logistic regression analysis of rs10758669 polymorphisms in patients with AAU and healthy controls stratified by AS and HLA-B27 status after adjustment for gender and age.

Model	Genotype	AAU					AAU+ AS+					HLA-B27(+)				
		control (n=710)	Case (n=923)	OR (95% CI)	P	Pc	Control (n=710)	Case (n=437)	OR (95% CI)	P	Pc	Control (n=710)	Case (n=535)	(95% CI)	P	Pc
Additive				1.09(0.93-1.27)	0.27	NS			1.07(0.88-1.28)	0.51	NS			1.15(0.97-1.37)	0.12	NS
Codominant	AA	346(48.7%)	405(43.9%）	Reference			346(48.7%)	185(42.3%)	Reference			346(48.7%)	220(41.1%)	Reference		
	CA	288(40.6%)	432(46.8%）	1.28(1.04-1.58)	0.02	NS	288(40.6%)	215(49.2%)	1.36(1.05-1.76)	0.02	NS	288(40.6%)	265(49.5%)	1.43(1.13-1.82)	3.50×10^{-3}	NS
	CC	76(10.7%)	86(9.3%）	0.97(0.69-1.38)	0.88	NS	76(10.7%)	37(8.5%)	0.84(0.54-1.31)	0.44	NS	76(10.7%)	50(9.4%)	1.01(0.68-1.52)	0.92	NS
Dominant	AA	346(48.7%)	405(43.9%)	Reference			346(48.7%)	185(42.3%)	Reference			346(48.7%)	220(41.1%)	Reference		
	CA+CC	364(51.3%)	518(56.1%)	1.22(1.00-1.49)	0.05	NS	364(51.3%)	252(57.7%)	1.25(0.98-1.60)	0.07	NS	364(51.3%)	315(58.9%)	1.35(1.07-1.69)	0.01	NS
Recessive	AA+CA	634(89.3%)	837(90.7%)	Reference			634(89.3%)	400(91.5%)	Reference			634(89.3%)	485(90.6%)	Reference		
	CC	76(10.7%)	86(9.3%)	0.86(0.62-1.20)	0.38	NS	76(10.7%)	37(8.5%)	0.72(0.47-1.10)	0.12	NS	76(10.7%)	50(9.4%)	0.85(0.58-1.25)	0.41	NS

OR = odds ratio; 95%CI = 95% confidence interval.

Pc = P value adjusted by Bonferroni correction.

Table S5. Multivariate logistic regression analysis of rs10975003 polymorphisms in female AAU patients and female controls after adjustment for gender and age.

		AAU(Female)				
**Model**	**Genotype**	**Control (Female) (n=643)**	**Case (Female) (n=334)**	**OR (95% CI)**	**P**	**Pc**
Additive				1.46(1.08-1.97)	0.01	NS
Codominant	TT	399(62.0%)	186(55.7%)	Reference		
	CT	216(33.6%)	135(40.4%)	1.57(1.11-2.23)	0.01	NS
	CC	28(4.4%)	13(3.9%)	1.59(0.64-3.96)	0.32	NS
Dominant	TT	399(62.0%)	186(55.7%)	Reference		
	CT+CC	244(38.0%)	148(44.3%)	1.57(1.12-2.21)	9.10×10^{-3}	NS
Recessive	TT+CT	615(95.6%)	321(96.1%)	Reference		
	CC	28(4.4%)	13(3.9%)	1.36(0.55-3.35)	0.51	NS

OR = odds ratio; 95%CI = 95% confidence interval.

Pc = P value adjusted by Bonferroni correction.
